# Systemic risk spillover between the stock market and banking deposits: Evidence from a sustainability perspective in the South Asian countries

**DOI:** 10.1371/journal.pone.0288310

**Published:** 2024-07-08

**Authors:** Linshan Liu, Amir Rafique, Naseem Abbas, Muhammad Umer Quddoos, Muhammad Munir Ahmad, Arslan Ahmad Siddiqi

**Affiliations:** 1 International Commerce Department, Concord University College, Fujian Normal University, Fuzhou, China; 2 Department of Management Sciences, COMSATS University, Islamabad, Islamabad, Pakistan; 3 COMSATS University Islamabad, Islamabad, Pakistan; 4 Department of Commerce, Bahauddin Zakariya University, Multan, Pakistan; 5 Department of Commerce, Allama Iqbal Open University, Islamabad, Pakistan; 6 Institute of Industrial and Control System, Karachi, Pakistan; University of Education, PAKISTAN

## Abstract

This research explores the link between stock markets and banking deposits in South Asian (Pakistan, India, Sri Lanka, Nepal) countries. This study empirically examines the systemic risk potential of financial institutions in South Asia using current systemic risk statistics. Yearly data on stock prices and banking deposits from January 2000 to December 2020 were analyzed using a two-stage process. In the first phase, we measure VaR (value at risk), and in the second step, we measure the DCC GARCH model for our empirical analysis. The study findings reveal systemic risk spillover between the stock markets of South Asian countries and the relevant country’s banking system deposits. The policymakers can use our study findings to create a more sustainable financial sector.

## 1. Introduction

Apart from the pension benefits, bank savings, and stock market investments are the two most valuable financial assets owned by families in, the United States of America and other nations (OECD, 2016). Consumers have moved their investment strategy among equities and deposits, directly impacting the quantity of money available to banks and the stock market and the financial activity of firms and people dependent on this kind of lending. Specifically, we demonstrate in this work that during stock market booms, individuals’ lower demand for deposits drains bank deposit financing, causing banks to shrink lending and, as a result, decreasing employment in bank-dependent industries [1]. This may affect the sustainability of financial institutions, where it has become a very crucial topic for many of the corporations, particularly financial institutions. Banks’ sustainability is incorporated in the traditional lending products which are dependent upon the banking deposits. Bank lending and deposits affect a bank’s performance, and the literature finds a positive association between bank performance and sustainability.

According to household portfolios, rebalancing could be a mechanism by which stock market fluctuations are transmitted to the banking sector. Carletti et al. [2] have theoretically and empirically discussed this idea. However, the practical significance of this theory has not been investigated, despite the huge body of research on the actual impacts of stock market swings [see e.g., 3, 4]. A comparable “deposit channel” for the transmission of monetary policy was recently proposed by [5]. It is easy to see how fluctuations in the federal funds rate influence the amount of money banks have on hand, affecting their lending and the rest of the real economy. Bank deposit funding and lending are negatively impacted by stock market shocks, which directly influence consumer demand for deposits.

Sustainability affects banks’ default risk and their systemic risk. When a financial institution has a crisis, transferring that crisis to another financial institution is commonplace in contemporary banking systems. The word “systemic risk” alludes to this tendency, which may result in systemic failure if it occurs on a large scale. Three types of systemic failure sources have been reported in academic literature. These are as follows: There is the potential for an “emergency bank run,” in which depositors and investors rush to withdraw their money, ultimately leading to the financial system’s collapse. An asset decline at one bank might have a detrimental effect on other banks’ solvency since banks invest in assets that are comparable to each other and own asset interlocking exposures among financial institutions, which, on the one hand, serve as a foundation for mutual insurance and, on the other hand, creates the possibility that the failure of one institution will have “blow consequences” on the economic condition of other institutions [6]. To manage that risk a sustainable banking model with higher sustainability scores is required, which is associated with lower default risk. The global financial crisis of 2007–2009 demands the sustainability of the finance industry [7]. Because of the global recession of 2007–2009, the study of systemic risk and its spillover in financial markets has recently received great attention in the finance literature. Extreme losses suffered by financial institutions have shown a propensity to spread across the whole financial system, resulting in system-wide instability and larger crises. Despite the global financial crisis and coronavirus (COVID-19) pandemic, the investment in the financial assets is increasing which has increased the need for understanding risk to achieve sustainability [8]. Understanding systemic risk is critical for investors and regulators, and the ability to predict its dynamics across parts and over time is critical for both.

Danger dispersion grows dramatically at times of high uncertainty in stock markets, putting the general maintainability of the global financial framework at risk of collapse [9–11]. Notably, negative occurrences may start a series of events, allowing gloomy expectations to propagate more quickly across the population. The World Bank, however, claims that the new coronavirus (known officially as COVID-19 and called “the most severe infectious health epidemic in history”) has hurt the stability of global financial markets, unlike other extreme events involving economic and financial factors (such as the 1997–98 Asian financial crisis; from 2007 to 2008, the global financial crisis; and from 2010 to 2013 European debt crisis) [12]. However, the risk of a global financial crisis continues to be a significant concern. The COVID-19 has created an additional strain on the banks’ liquidity, increasing the risk of default and insolvencies on a larger scale thus affecting the sustainability of the whole financial sector.

To put it another way, systemic risk has recently taken center stage in global economic discourses following the unpleasant experience of the 2007 subprime mortgage crisis [13, 14]. During the Great Recession, the financial system became overburdened due to distress and, in some instances, the collapse of critical institutions, resulting in increased hardship and the real-world consequences of economic shocks. The inability of regulators, entrusted with the responsibility of ensuring the financial system’s health, to take effective measures to manage systemic risk is due to a lack of understanding of the disclosure and ability of appropriate stakeholders to contribute to systemic risk in the financial system. This also demands a strategy to both protect banks and their members for a resilient financial sector.

Even as the integration of financial markets continues to grow quickly, regulators and supranational organizations are becoming more concerned about the possibility of catastrophic failure in the banking industry. The primary risk is that the bankruptcy of banks simultaneously would result in a major economic catastrophe of unprecedented proportions. In the past, we have seen that the economic consequences of a financial crisis may be severe, as we have already seen with other crises. For example, according to [15], production declines by an average of 15–20 percent of GDP during a financial crisis. In order to avert another financial crisis, the International Basel Committee introduced new capital and liquidity standards in December 2009 for the banking sector. The reforms were known as Basel III and came into force with effect from November 2010. These standards have increased the soundness and sustainability of the banking sector worldwide. During the 12 years ending in 2017, the South Asian area continued to sustain an average yearly expansion of economic progress of almost 7 percent (World Bank 2017). According to the World Bank’s 2016 financial report, South Asia is the world’s fastest-growing region in terms of economic growth. The area is predicted to retain its strong development momentum throughout 2019 to 2020 by boosting the average yearly growth rate to 7.1 percent. The establishment of liberalized economic policies by the member nations of the area is the driving force behind this exceptional pace of economic development. The liberalization of financial markets has played an important part in this process. Since the 1980s, South Asian countries have deconstructed their regulatory frameworks in response to financial help from the International Monetary Fund (IMF), the World Bank, and the Asian Development Bank (ADB). The ultimate goal is to increase the competitiveness of the financial services industry. The McKinnon–Sear model as proposed by [16] argues for increased government engagement in financial market operations while pushing for the liberalization of financial market activity in emerging nations to increase economic progress. Because of the liberalization of financial systems, market discipline has been formed. It has been suggested that it may assist financial institutions in reducing equity and credit risks they face while operating in a very strictly regulated environment [17]. The regulatory framework can be extended in different directions to cater to diverse types of financial risks and to mitigate the contagion of these risks in financial markets to develop a sustainable financial sector. As a result, the current research seeks to address this vacuum by adding to the existing body of literature in two ways: by contributing to the existing body of literature; and by contributing to the existing body of literature. First, most of the empirical research on these metrics has been conducted in developed markets, especially in Europe and the United States, with few exceptions. In contrast to the United States, where the subprime crisis and Great Financial Crisis started, the European sovereign debt crisis, which began in 2010, has its beginnings in Europe. It means that current methodologies are being extensively evaluated or deployed in the context of institutions from these two areas, which is expected given their proximity.

### 1.1 Research gap and study significance

Investors from all around the world are paying attention to developing Asian stock markets. The South Asian market has seen rapid expansion in recent decades. Markets are under the impact of regional, domestic, and world dynamics. Policymakers are constantly trying to ensure financial stability while preventing and mitigating the effects of financial crises. Numerous policy efforts have been implemented since the start of the global financial crisis. It is useful in reducing systemic risk. In this study, we are looking at banks’ deposits, which influence the performance of the stock markets, which is why this problem must be addressed and explored. The relationship between deposits and stocks is affected by the stock market movement and vice versa. Bank deposits and lending patterns affect the stocks of bank-dependent firms, so the systemic risk affects not only the banking sector but also the firms that are dependent upon the banking sector.

There have been conducted various studies examining the spillover effects of the bank runs upon the different sectors and stock returns in the economy [see e.g., 18] examining the Spillover effects of US monetary policy on banking development: evidence from the Asian region [19, 20]; examining the Liquidity, bank runs and bailouts: spillover effects during the Northern Rock episode [21, 22]; examining the spillover effects of sovereign rating changes [20]; examining the Spillover effects between Greece and Cyprus [23]; and examining the Interest rate and bank stock returns asymmetry [24].

Notably, regarding Asian institutions, quite a few studies [e.g., 19, 25] have looked at systemic risk and financial fragility in Asian countries. However, none deals with the problem in a regional context; instead, the studies that have been done thus far have concentrated on individual economies, and that too disregarding the banking deposit growth. This study employs the data from the Asian countries to understand the impact of risks on the financial sector and the banking deposits.

Second, in methodology, we apply two relatively new techniques to the literature: the value-at-risk approach and the DCC GARCH [26], which developed the concept of the coefficient as a systemic risk metric. By providing information on the market’s value-at-risk (VaR), when a market is linked with an institution suspected of being in a financial crisis, it can assess the ability for systemic risk spillover between the two [27, 28]. Quantile regression is used to obtain CoVaR estimates in this study as quite a few researchers have attempted to compare the CoVaR among various market segments. For example, [29] investigated systemic risk spillovers among currency and stock markets in developing regions. The CoVaR risk model is utilized to model systemic risk in the European banking sector [30] to predict systemic risk in the European banking industry, while [31] employs CoVaR to simulate the conditional tail risk of cryptocurrencies when seen in the context of global financial assets.

The spillover of the risk between banking deposits and stock markets is very important to have a sustainable financial sector. This research examines the systemic risk spillover between the stock markets of South Asian countries and their banking deposits. Therefore, the objective of the study is as follows:

1. To analyze systemic risk spillover between the stock markets of South Asian countries and their banking deposits.

This research will benefit financial investors, portfolio managers, academic institutions, and financial institutions. Furthermore, it is advantageous for policymakers, who may utilize knowledge regarding the systemic risk spillover of the South Asian stock markets and banking deposits to formulate sound policies for a sustainable financial sector. We are also employing value at risk (VAR) and conditional value at risk (CoVar) to analyze the systemic risk spillover between the stock market and banking deposits in South Asian nations, a new concept.

## 2. Literature review

Portfolio theory, often known as modern portfolio theory (MPT), is a rational approach for selecting securities to maximize their overall returns while minimizing risk to a reasonable level. Using portfolio theory has the primary benefit of allowing for the consideration of correlations across stress indicators, both inside and across sectors of the economy [32], as in the case of this study for deposits and stock markets.

### 2.1 Systemic risk and banking deposits

Systemic risk occurs due to a singular occurrence that has wide-ranging consequences for the whole sector and may even cause the whole economy to collapse [33–35]. When we speak about this risk in finance, we refer to it as the collapse of the financial sector, and since it is linked to the financial sector, it has the potential to lead the economy to recession. If we look back at recent history, we can see how systemic risk manifested itself in the global financial crisis of 2008, as well as in the case of COVID-19. Systemic risk may be described as a danger that affects the whole body and is exacerbated by minor problems. These risks have increased the interest of the researchers to study the spillover of risk and to identify the strategies to have a sustainable financial sector.

The researchers examined the systemic risk posed by banks from 2004 to 2012, as well as in the United States and the European Union. According to their findings, the size of the banks is essential in evaluating systemic risk. They can obtain new metrics that are superior predictors of financial difficulty during the subprime mortgage crisis from 2007 to 2009. They have concluded that the new measurements might be beneficial when combined with current indicators [14]. According to their findings, financial hardship is a situation in which a bank’s assets are less than its liabilities in the future. The gap between assets and liabilities of the banking sector should not be too much so this can negatively affect the stability of the banking sector.

In an earlier research [36], the authors gathered systemic risk emanating from several sources and then constructed measures using data from the twenty largest enterprises in three countries: the United States, Britain, and the European Union. The findings of this research demonstrate how systemic risk and financial market collapse impact the distribution of economic activity. It is also hypothesized that changes in the 19 distinct measures of distribution would cause a shock to the industry’s output and other variables in the United States and the European Union. According to their findings, systemic risk measures provide more information about shocks in the macroeconomic left tail than in the macroeconomics’ right tail and the central tendency measure. They also demonstrate that the financial sector’s equity volatility contributes to macroeconomic downturns.

Bisias et al. [37] surveyed thirty-one systemic risk metrics in the fields of economics and finance chosen to span the key issues and themes in connection with systemic risk measurement and management.

To determine the gap, [38] use two methodologies. It is the first in which they used primary data to create a systemic danger in isolation. The second technique used secondary or market data to develop global metrics not tied to a single theoretical framework. A bank’s failure has a detrimental impact on other banks still in business, causing a recession spillover. They also address how a systemic event must occur such that banks are not just connected but also huge enough to be systemically important. The systemic risk, if not identified and managed properly, can affect the sustainability of the whole financial sector.

In another study [39], the authors investigated the influence of bank diversity on systemic risk. Individual banks benefit from diversification because it boosts their ability to respond to shocks timely. They examine the diversification of banks using data from commercial banks in the United States from 2000 to 2013. It is discovered that the influence of diversification on systemic risk is considered in the case of large and medium-sized financial institutions. The researchers also discovered that diversification substantially impacted the financial crisis of 2007–2009 and the European debt crisis from 2010 to 2013. Regarding systemic risk, banks’ diversity is critical for the success (Yang et al., 2020) and soundness of the banking sector.

### 2.2 Systemic risk and stock market

For the period from 2009 to 2018, [40] found significant interindustry volatility spillover in the Shanghai Stock Exchange. The spillover index becomes more important when there is a bull market and a down market. [41] investigated systemic risk spillover and dependency between oil indices and stock markets in G-7 countries (Canada, France, Germany, Italy, Japan, United Kingdom, and the United States) using data from January 2003 to November 2017. During this period, several financial and energy crises occurred simultaneously. They use time-varying, time constant, and time-varying Markov copula models to look at the relationship between the stock market and oil prices. They discovered that the returns of the oil series are not related to the stock markets of France, Germany, and Japan from G-7 countries but are dependent on the stock markets of Canada and the United States. The relationship with the United Kingdom is stable, but the relationship with Italy is nearly nonexistent. While the Canadian stock market is sensitive and dangerous, it is also susceptible to negative impacts from the market for crude oil when compared to other markets in the world. According to their findings, investors should consider crude oil market prices as a good diversifier for Japan and France but not for other G7 countries [41].

Furthermore, another study [42] explores the effects of oil price shocks on the instability of the stock market in the United States, and other G7 economies. They discovered, among other things, that swings in oil prices had little influence on the stock market’s volatility. The study [42] employed dynamic conditional correlation (DCC) to explore the link between the fluctuation of oil prices and industrial commodity prices on the S&P 500 index. Additionally, commodities experience volatility in response to economic and global disasters, while the S&P-500 experiences volatility in response to economic and global meltdowns. The closing prices of goods (WTI oil, Brent oil, Cooper, Silver, and Gold) and the closing prices of the S&P-500 are used in this study. They found that the prices of all five commodities and the S&P-500 are subject to high and low volatility.

In another study [43], the authors looked at the volatility of the returns on the Pakistani stock market and the foreign currency market. They used the Engle-Granger approach to examine long-run connections and the bivariate EGARCH model to investigate the impact of volatility spillover. According to the study results, there is no long-term association between the two markets. They concluded that the behavior of both markets is intertwined and that the foreign exchange market’s volatility influences the returns on stocks. Their analysis also demonstrated a substantial association between the volatility of the foreign currency market and the volatility of returns of the stock market of Pakistan.

Another study [44] analyzed the relationship between the stock markets of developed countries (the United States and Japan) and the stock markets of emerging countries in Asia (China, India, Indonesia, Malaysia, the Philippines, and Bangladesh) from 1993 to 2012. According to their findings, there was a one-directional disturbance and instability spillover from the US market to developing Asian markets and the developed market of Japan. They also discovered that during the global financial crisis of 2008, the volatility spillover in the stock markets of the United States and Asia was larger and more bidirectional than in previous years. They also discovered that during the previous five years, a significant relationship had emerged between the Japanese stock market and the growing markets of Asia. Furthermore, rather than focusing on return spillover, they focused on shocks and volatility spillover [44].

A considerable influence of COVID-19 on the stock market has been shown in the literature; thus, [45] investigated the shift in volatility from lower to greater levels using a Markov switching AR model during the COVID-19 period. Their findings indicated that economic indices influence fluctuation and that volatility is especially responsive to news about COVID-19. Both negative and positive COVID-19 news are statistically significant; however, negative COVID-19 news is statistically much more significant. COVID-19 has led to exceptional fluctuations in the financial markets of the United States of America. In each industry, a rise in overall and individual risks is noticed [45]. The spillover of volatility from one market to another market or from one asset to another asset can increase the volatility of the whole financial sector thus severely affecting the sustainability of all the financial instruments, financial markets, and financial institutions [1, 44, 46].

### 2.3 Stock market and banking deposits

Banking deposits are sums of money that depositors have in excess and want to preserve for future investment or consumption. Banks utilize deposits to make money by lending it to other people. Banking deposits may be divided into three categories: current account, savings account, and fixed account. Bank deposits show the financial stability of a bank.

Another study [47] assessed the deposits available in the Islamic banking sector. Banks need a consistent flow of deposits to maintain their financial stability. Islamic banks, unlike conventional banks, are required to comply with the standards of Islamic Shariah (Islamic law). According to their findings, deposits are both demand-elastic and supply-elastic in terms of returns on deposits. Other variables, such as the rate of inflation, the size of the money market, and the degree of awareness, have a considerable impact on the supply of deposits and the profitability of banking, while the growth of Islamic finance has a substantial effect on the demand for deposits. According to this research, deposits in Islamic banking are steady and well-connected with one another. They recommend that banks check their pricing relationship while developing a plan to attract new consumers to the bank [47].

In another study [48], the authors investigated the determinants of bank deposits in Ghana by using a random effect technique and data from 2008 to 2017. The factors were classified into two categories: microeconomic variables and macroeconomic elements. The microeconomic factors are those tied to banks and individual variables, while the macroeconomic elements are those related to the economy. According to their findings, the size of the bank, its liquidity, and its profitability were the most important variables influencing banking deposits. Inflation has a negative and statistically significant impact on bank deposits. Furthermore, their findings demonstrated that increased capital does not inevitably result in higher deposits. The size of the bank is crucial for attracting deposits [1, 2, 49].

When the stock market is booming, bank deposits drop significantly. As a result of a decline in deposits, banks reduce their lending, which has a detrimental effect on investment and the economy. Depositors either invest in banks or the stock market when they want to obtain a high rate of return on their investments. When the returns on stocks are higher, banks will notice a significant decline in deposits, particularly in areas where individuals have a larger level of stock ownership than in other areas. Because of the strong returns on stock, there has been a fall in the number of depositors, which has resulted in a decrease in bank lending [1]. When investors change asset allocation between deposits and stocks, for any reason, this affects the funding available for banks and the stock markets, which are traditional investment instruments. Being traditional instruments, these are highly correlated and the chance of spillover of risks is too high in these markets. Identifying and managing the spillover of systemic risk is necessary for the sustainability of the financial sector, as the banking sector and stock markets play a crucial role in the growth and stability of the financial sector. Due to household portfolio rebalance the fluctuation can easily be transmitted from one sector to the other sector. Portfolio rebalancing plays a crucial role in the stability of the financial sector. Several variables including the base lending rate, consumer price index (CPI), Karachi Inter-bank Offer Rate KIBOR, money supply, gross domestic product (GDP), the Karachi Stock Exchange (KSE) composite index, and the profit rate of Islamic banking, were examined in the study. Akhtar et al. [50] used quarterly data from 2006 to 2011 of thirty banks, which include five Islamic banks and twenty-five conventional banks. They examined long-term relationships and short-term relationships using the advanced time series technique. They found that factors such as the profit of Islamic banks, the interest rate of a conventional bank, the money supply, based lending, and consumer prices significantly impact the deposits of Islamic banks and conventional banks.

In another study [51], the authors looked at the significant changes in operational technology and ownership structure that have occurred in recent years and how they have affected the efficiency of Chinese banks. An empirical examination of operational data from seventy-one conventional banks in China over five years (2011–2015) was made in the study. The study reveals that the kind of ownership and the owners’ concentration impact the deposits’ efficiency, while the efficiency of loans is influenced by the liquidity and type of ownership in the business [51].

The impact of the global financial crisis and macroeconomic factors on the deposits of Islamic banks in Malaysia is investigated in the research [52]. They used monthly data spanning the period from January 2000 to December 2010. They employed the cointegration test and the vector error model to uncover the dynamic link between the financial crisis and macroeconomic factors, as well as deposits in Islamic banking institutions. According to their findings, no statistically significant relationship exists between changes in interest rate, profit rate, or output growth. Inflation hurts Islamic banking deposits. Surprisingly, the financial crisis had a favorable impact on deposits in Islamic financial institutions. During the financial crisis, depositors placed their faith in Islamic banks, believing they would be more flexible. The influx of deposits into Islamic banking during the financial crisis of 2007–08 was found in the study [52].

## 3. Data and methodology

The study uses data from banking deposits and stock markets of South Asian countries (Pakistan, India, Nepal, and Sri Lanka). Our data sample consists of banking deposits and the stock market from January 2001 to December 2020. For deposits, we take the bank deposits to GDP ratio previously taken by [53]. The data on stock prices is gathered from the Yahoo Finance website and the of banking deposits is gathered from the World Development Indicator (WDI) website. For deposits, we are taking bank deposits to GDP ratio previously taken by [54].

A bank is a type of financial intermediary organization that connects the surplus and deficit groups to keep production going and finance other economic activities [52]. According to [55], indirect financing, which involves the actions of financial brokers, is far more crucial to economic growth than direct financing, which involves businesses getting money from lenders directly on the financial markets. For the years 1970 through 1996, for instance, bank loans accounted for 85% of the external funding for nonfinancial enterprises in Japan and financial markets accounted for 15%, whereas in Germany it was approximately 80% from bank loans and the remaining from financial markets [55]). Banks, however, require cash inflow to expand the operations of their financial intermediaries and enhance their contributions to economic growth. Money from shareholders alone is insufficient. To provide loans or financing to boost economic growth and productivity while also making a profit on their own through applied interest or margin, banks need money from the general public as an inflow. Deposits are crucial for banks and a nation’s economy because of this [52]. Banks act as middlemen in the mobilization of savers’ cash, which is subsequently lent to private and public investors. Without question, deposits are the foundation of commercial banks’ operations. By description, bank deposits are sums of money that consumers put into commercial banks for both safekeeping and the opportunity to earn interest.

In theoretical terms, stock markets form the core of financial structures. Stock markets serve as a means for converting savings into funding for the real economy, which is their main purpose. Theoretically, stock markets can spur economic growth by enhancing domestic savings, increasing investment volume and quality, and mobilizing domestic savings. Better savings mobilization could boost saving rates, and if stock markets direct savings towards investments that will generate higher returns, the rate of interest to savers will rise, making savings more alluring. Therefore, the corporate sector will receive more savings. Enterprises compete on an even playing field for capital-inefficient stock markets, which also contributes to more effective investment. Stock markets have many other crucial roles in the economy in addition to this one [56].

### 3.1 Sample

In following Table 1, the stock market indices are presented that are used in the study:

**Table 1:** Sampled stock markets.

Countries	Index
Pakistan	PSX
India	NSE
Sri Lanka	CSE
Nepal	NSE

We selected this sample since several studies are available on the European and American stock exchanges [41, 42, 57], but very few studies are available on the South Asian stock exchanges. The stock markets in South Asia have undergone considerable development over the past many years. Even throughout periods of global economic downturn, these stock exchanges have shown remarkable endurance for remaining stable.

The study uses data from the South Asian stock markets and the country’s bank deposits from January 2001 to December 2020. This data span is important for our study because this period covers three main shocks: the global financial crisis of 2007–08, the European debt crisis of 2010–2013, and COVID-19. The spread of COVID-19, the implementation of movement restriction policies, and the emergence of uncertainty in the global economy all harmed the sustainability of the stock markets in the majority of countries [58].

### 3.2 Methodology

In this chapter, we discuss two measures to find the spillover and systemic risk between the banking deposits and stock markets of South Asian countries. First, we use VaR (value at risk) to find the spillover between banking deposits and stock markets, and second, the study calculates coefficients to find the systemic risk spillover between banking deposits and stock markets. The data on stock prices is gathered from the Yahoo Finance website and the of banking deposits is gathered from the World Development Indicator (WDI) website. For deposits, we are taking bank deposits to GDP ratio previously taken by (Mushtaq & Siddiqui, 2017).

There are three types of bank deposits- current deposits, savings deposits, and term deposits. Current deposits are the type of money that is deposited by an individual with the bank, and the depositary may take it out whenever he appeals (Schonharl, 2017). Current deposits, which banks are required to pay, are also known as demand deposits. Savings deposits are contracts between a client and a bank in which the customer deposits money and receives interest, with the customer’s ability to withdraw funds from the account at any time without giving notice [59]. With term deposits, the bank has the freedom to allocate its capital with the understanding that the money would not be available for cheque withdrawal (Mishkin, 2004).

By enabling long-term investments to be financed through individual funds, those who want the flexibility to withdraw their assets whenever they choose stock markets can execute an "act of magic" (Baumol, 1960). Furthermore, by fostering competition across various categories of financial products, stock markets can improve the effectiveness of the financial system. This can therefore reduce the cost of borrowing money for borrowers and increase the return on investments for those who save. Accounting and tax rules may also be improved by stock markets as investors seek out greater and superior information to evaluate the performance of various companies. Thus, it makes sense that supplying such information would be in the company’s best interest to enable in-depth comparisons between rival companies. Being sensitive to changes in policy, especially monetary policy, financial markets by their very presence helps strengthen policy creditability. This is one of the many significant benefits of the presence of stock markets: the possible imposition of more discipline in the field of economic management. Several listed companies and market capitalization are the two indicators of the stock market which measure the size of the stock market [56].

#### 3.2.1 Step one (VaR)

First, we construct the unconditional value at risk (VaR) model for the stock markets and banking deposits of the countries in South Asia. For this paper, we assume that every return series is represented by the first order of an autoregressive AR (1) model in the following way:

$$R_{it}=C_{i}+v_{i}r_{it-1}+\epsilon_{it},\varepsilon_{it\lfloor {Ω}_{t-1}\approx N(0,H_{t}\rfloor }$$ (1)

where *r*_*it*_ denotes the returns of the stock markets and banking deposits of South Asian countries; *r*_*it*−1_ is the autoregressive return, which adjusts for possible cointegration; *c*_*i*_ is continual; and *v*_*i*_ is the component of autoregressive returns. *e*_*it*_ is the error term that is based on previous data Ω_t−1_ is at a time (t–1).

$$h_{it}=w_{i}+\alpha_{i\epsilon_{t-1}^{2}}+\beta_{i}h_{it-1},$$ (2)

where the conditional variance of every return series is denoted by *h*_*it*_, *w*_*i*_ is a constant that evaluates unconditional volatility; *α*_*i*_ captures the stock market impact; and *β*_*i*_ captures the impact of the deposits.

A return *r*_*i*_ of the stock market or banking deposits at time t with a confidence level of q, VaR, is defined as the *q*^*th*^ quantile of the return distribution [60]:

$$p(r_{t}^{i}\leq {VAR}_{t}^{i,q})=q$$ (3)

We calculate VaR for the stock market and banking deposits as follows:

$${VAR}_{q,t}^{i}=\phi^{-1}(q)\sigma_{t}^{i}$$ (4)

#### 3.2.2 Step two (DCC GARCH)

We determine CoVaR, CoVaR^*g*/*i*^ for stock market g and bank deposits (i) at time t as the q-quantile of the conditional distribution. This measure is defined as follows:

$$p\left(r_{t}^{g}\leq {CoVaR}_{t}^{\frac{g}{i},q}/r_{t}^{i}\leq {VaR}_{t}^{i,q}\right)=q$$ (5)

Based on Eq (5), we proceed to calculate ΔCoVaR^*g*/*i*^, ‘‘contribution” CoVaR, to evaluate the stock market conditional VaR when bank deposits (i) move from a normal state to a state of distress [26]:

$$\Delta {CoVaR}_{t}^{g/i,q}={CoVaR}_{t}^{g/i,q}-{CoVaR}_{t}^{g/b^{i},q}$$ (6)

where *b*^*i*^ represents the country’s deposits (normal) state.

$${CoVaR}_{t}^{\frac{g}{i},q}=\phi^{-1}(q)\sigma_{t}^{g}\sqrt{1-p_{gi,t}^{2}}+\phi^{-1}(q)p_{gi,t}\sigma_{t}^{g}$$ (7)

where *p*_*gi*,*t*_ is the conditional correlation between the stock markets and deposits of countries, *σ*^*g*^ is the stock market conditional standard deviation, and q is a confidence level equal to 5%. Moreover, we calculate the stock market ΔCoVaR^g/i^ to evaluate bank deposits’ marginal risk contribution to stock market unconditional risk. This measure can be calculated as the difference between the stock market superscription ΔCoVaR^g/i^ when a bank deposit is (or is not) in distress [60].

## 4. Data analysis and results

This section presents the analysis of data output and discussion thereof as follows:

### 4.1 Descriptive statistics

Table 2 above shows the descriptive statistics of the data (mean, median, maximum, minimum, standard deviation, skewness, kurtosis, Jarque-Bera). The mean of Indian bank deposits is 62.38501, the standard deviation is 6.917401, and the mean and standard deviation of the Indian stock exchange are 5656.141 and 3419.029, respectively. The mean of Nepal bank deposits is 65.2342, the standard deviation is 21.55547, and the mean and standard deviation of the Nepal stock exchange are 728.1412 and 470.7273, respectively. The mean of Pakistan bank deposits is 32.55398, the standard deviation is 2.497351, and the mean and standard deviation of the Pakistan stock exchange are 19135.39 and 14779.14, respectively. The mean of Sri Lankan bank deposits is 37.22654, the standard deviation is 11.06372, and the mean and standard deviation of the Sri Lankan stock exchange are 4130.169 and 2371.206, respectively. Kurtosis is acceptable, with a value between -3 and +3, and the values of our variables all lie between -3 and +3. The kurtosis value is acceptable if it lies between -10 and +10, and the values of our variables lie between -10 and +10. Jarque-Bera shows the normality of variables; in the given study, all the variables are normally distributed.

**Table 2:** Descriptive statistics.

	INDIA_BANK_DEPOSITS	NEPAL_BANK_DEPOSITS	PAKISTAN_BANK_DEPOSITS	SRI_LANKA_BANK_DEPOSITS	NIFTY_50	NEPSE	KSE_100	CSE
**Mean**	62.39	65.23	32.55	37.23	5656.14	728.14	19135.39	4130.17
**Median**	65.05	64.87	32.58	31.76	5365.25	597.99	12351.62	5256.95
**Maximum**	74.97	103.11	36.91	59.69	11535.40	1548.54	45135.94	7104.78
**Minimum**	47.71	38.74	26.66	27.10	1045.48	207.28	1331.53	455.38
**Std. Dev.**	6.92	21.56	2.50	11.06	3419.03	470.72	14779.14	2371.21
**Skewness**	-0.56	0.27	-0.44	1.09	0.30	0.60	0.50	-0.24
**Kurtosis**	2.58	1.74	2.97	2.45	1.95	1.95	1.71	1.41
**Jarque- Bera**	1.18	1.56	0.64	4.20	1.20	2.11	2.22	2.31

### 4.2 Correlation matrix

Table 3 above shows the correlation between the variables. Correlation values of 1 show that the variables are perfectly positively correlated, values between 0.8 and 1 show that the variables have strong positive correlations, and values between 0.6 and 0.8 show that the variables are moderately correlated. The values from 0.4 to 0.6 show that the variables are weakly correlated, and values from 0 to 0.2 show that the variables have strongly weak correlations.

**Table 3:** Correlation matrix.

	INDIA_BANK_DEPOSITS	NEPAL_BANK_DEPOSITS	PAKISTAN_BANK_DEPOSITS	SRI_LANKA_BANK_DEPOSITS	NIFTY_50	NEPSE	KSE_100	CSE
**INDIA_BANK_DEPOSITS**	1.00	0.83	0.35	0.46	0.79	0.70	0.70	0.82
**NEPAL_BANK_DEPOSIT**	0.83	1.00	0.37	0.85	0.97	0.89	0.93	0.83
**PAKISTAN_BANK_DEPOSITS**	0.35	0.37	1.00	0.52	0.45	0.50	0.48	0.15
**SRI_LANKABANK_DEPOSITS**	0.46	0.85	0.52	1.00	0.86	0.85	0.89	0.57
**NIFTY_50**	0.79	0.97	0.45	0.86	1.00	0.88	0.95	0.84
**NEPSE**	0.70	0.89	0.50	0.85	0.88	1.00	0.93	0.64
**KSE_100**	0.70	0.93	0.48	0.89	0.95	0.93	1.00	0.80
**CSE**	0.82	0.83	0.15	0.57	0.84	0.64	0.80	1.00

### 4.3 ARCH and DCC GARCH for Indian stock market and bank deposits

Table 4 presents the heteroskedasticity test of the ARCH model, whose coefficients are 9.94 and 0.29, and both positively impact current conditional variance. The p-value is 0.04, which is significant. The p-value of the residual is 0.14, which is more than 0.1 and is insignificant. The value for F-statistics is 0.14.

**Table 4:** ARCH and DCC GARCH for Indian stock market and bank deposits.

Heteroskedasticity Test: ARCH				
Variable	Coefficient	Std. Error	t-Statistic	Prob.
**C**	9.94	4.56	2.17	0.04
**RESID^2(-1)**	0.29	0.19	1.51	0.14
**Prob(F-statistic)**	0.14			

#### 4.3.1 DCC GARCH

Table 5 above shows the DCC GARCH model results in which the coefficient value of theta (1) positively impacts conditional dependent volatility. The p-value of theta is less than 0.1, which is 0.07; thus, we can say it is significant. The coefficient value of theta (2) also positively impacted conditional volatility, and the p-value of theta (2) is also less than 0.1, which is 0.002, and we can say it is significant. The stability condition that theta (1) + theta (2) < 1 is met indicates that the model is correct, and the volatility of the stock market of India has a significant positive impact on the dynamic relationship with Indian banking deposits.

**Table 5:** DCC GARCH.

Estimation Method: ARCH Maximum Likelihood (BFGS)—Two-Step				
	Coefficient	Std. Error	z-Statistic	Prob.
**theta (1)**	0.30	0.16	1.79	0.07
**theta (2)**	0.61	0.20	3.04	0.002
* Stability condition: theta (1) + theta (2) < 1 is met.				

### 4.4 ARCH and DCC GARCH for Nepal stock market and bank deposits

The results presented in Table 6 show that the coefficients are 61.83 and 0.29, and both positively impact current conditional variance. The p-value is 0.05, which is significant. The p-value of the residual is 0.22, which is insignificant. The value of the F-statistic is 0.22.

**Table 6:** ARCH and DCC GARCH for the Nepal stock market and bank deposits.

Heteroskedasticity Test: ARCH				
Variable	Coefficient	Std. Error	t-Statistic	Prob.
**C**	61.83	30.33	2.03	0.05
**RESID^2(-1)**	0.29	0.23	1.26	0.22
**Prob(F-statistic)**	0.22			

#### 4.4.1 DCC GARCH

The DCC GARCH model results in Table 7 show that the coefficient value of theta (1) positively impacts conditional volatility. The p-value of theta (1) is less than 0.1, which is 0; thus, we can say it is significant. The coefficient value of theta (2) hurts conditional volatility, and the p-value of theta (2) is also less than 0.1, which is 0; thus, we can say it is significant. The stability condition that theta (1) + theta (2) < 1 is met says that the model is correct, and the volatility of the stock market of Nepal has a significant positive impact on the dynamic relationship with the Nepal banking deposits.

**Table 7:** DCC GARCH.

Estimation Method: ARCH Maximum Likelihood (BFGS)—Two Step				
	Coefficient	Std. Error	z-Statistic	Prob.
**theta (1)**	0.74	4.60	1.61	0
**theta (2)**	-0.12	7.02	-1.84	0
* Stability condition: theta (1) + theta (2) < 1 is met.				

### 4.5 ARCH and DCC GARCH for Pakistan stock market and bank deposits

The results presented in Table 8 show a coefficient of 4.14, which is more than 0 and positively impacts the current conditional variance; the coefficient of -0.08 is less than 0 and harms the current conditional variance. The p-value is 0.008, which is significant. The p-value of the residual is 0.65, which is insignificant. The value for F-statistics is 0.65.

**Table 8:** ARCH and DCC GARCH for Pakistan stock market and bank deposits.

Heteroskedasticity Test: ARCH				
Variable	Coefficient	Std. Error	t-Statistic	Prob.
**C**	4.14	1.39	2.97	0.008
**RESID^2(-1)**	-0.08	0.19	-0.45	0.65
**Prob(F-statistic)**	0.65			

#### 4.5.1 DCC GARCH

The DCC GARCH model results presented in Table 9 above show that the coefficient value of theta (1) positively impacts conditional dependent volatility. The p-value of theta is less than 0.1, which is 0.00; thus, we can say it is significant. The coefficient value of theta (2) is less than 0 and harms conditional volatility, and the p-value of theta (2) is also less than 0.1, which is 0.00, and we can say it is significant. The stability condition that theta (1) + theta (2) < 1 is met says that the model is correct, and the volatility of the stock market of Pakistan has a significant positive impact on the dynamic relationship with the Pakistan banking deposits.

**Table 9:** DCC GARCH.

System: 2-Step DCC (1,1) model with univariate GARCH fitted in the 1st step				
Estimation Method: ARCH Maximum Likelihood (BFGS)—Two Step				
	Coefficient	Std. Error	z-Statistic	Prob.
**theta (1)**	0.72	2.35	3.08	0
**theta (2)**	-0.18	5.23	-3.57	0
* Stability condition: theta (1) + theta (2) < 1 is met.				

### 4.6 ARCH and DCC GARCH for Sri Lanka stock market and bank deposits

The heteroskedasticity test results of the ARCH model presented in Table 10 above show that the coefficients are 1.60, which is more than 0 and has a positive impact on the current conditional variance, and 0.47, which is also more than 0 and has a positive impact on the current conditional variance. The p-value is 0.15, which is insignificant. The value for F-statistics is 0.03.

**Table 10:** ARCH and DCC GARCH for Sri Lanka stock market and bank deposits.

Heteroskedasticity Test: ARCH				
Variable	Coefficient	Std. Error	t-Statistic	Prob.
**C**	1.60	1.08	1.48	0.15
**RESID^2(-1)**	0.47	0.21	2.25	0.03
**Prob(F-statistic)**	0.03			

#### 4.6.1 DCC GARCH

The DCC GARCH model results presented in Table 11 show that the coefficient value of theta (1) positively impacts conditional dependent volatility. The p-value of theta is more than 0.1, which is 0.11; thus, we can say it is insignificant. The coefficient value of theta (2) is less than 0 and harms conditional volatility, and the p-value of theta (2) is also more than 0.1, which is 0.23, and we can say it is insignificant. The stability condition that theta (1) + theta (2) < 1 is met indicates that the model is correct, and the volatility of the stock market of Sri Lanka has a significant positive impact on the dynamic relationship with Sri Lankan banking deposits.

**Table 11:** DCC GARCH.

Estimation Method: ARCH Maximum Likelihood (BFGS)—Two Step					
	Coefficient		Std. Error	z-Statistic	Prob.
**theta (1)**	0.19	0.12		1.56	0.11
**theta (2)**	-0.69	0.57		-1.19	0.23
* Stability condition: theta (1) + theta (2) < 1 is met.					

The negative correlation between theta 1 and theta 2 of Pakistan, Bangladesh, and Sri Lanka indicates the inverse relationship between the variables of these countries which means when there is an increase in the stock market there will be a decrease in deposits and vice versa. The positive correlation of theta 1 and theta 2 of India indicates the direct relation between the stock market and banking deposits of India.

## 5. Conclusion and policy implications

In this study, we use the Nifty 50, NEPSE, KSE 100, and CSE stock prices to find the systemic risk spillover on the banking deposits of the South Asian countries. This study aims to find the systemic risk spillover between the stock markets and the banking deposits of South Asian countries. We employ the DCC GARCH model to achieve the objective. There are four Asian stock markets and banking deposits used in the study (India, Nepal, Pakistan, and Sri Lanka). The study establishes the presence of a systemic risk spillover between the stock markets and the banking deposits of South Asian countries. The results show a significant positive impact between the stock market of India and their banking deposits. The results of Nepal and Pakistan show that theta (1) has a positive significant impact and theta (2) has a significant negative impact between stock markets and banking deposits. The results for Sri Lanka show that theta (1) positively impacts the relationship between the stock market and banking deposits. The theta (2) shows the insignificant negative impact between the stock market and banking deposits. If we look at the result of DCC GARCH, which has a stability equation of stability condition: theta (1) + theta (2) < 1. The stability condition in all countries is present in the DCC GARCH model.

This paper finds the association between the bank deposits and stock markets. This association increases with the spillover of systemic risk. The effect is stronger where the financial sector is highly dependent upon these two sectors. The transmission of risk not only affects these two sectors but also affects the real economy. These two sectors are interrelated in terms of volatility spillover, thus affecting the sustainability of the whole financial sector. Due to the transmission of fluctuations in the stock market through the deposit channel, the firms that are equity and lending-dependent are more likely to be financially constrained which is detrimental to the sustainable financial sector.

In this study, we use the Nifty 50, NEPSE, KSE 100, and CSE stock prices to find the systemic risk spillover on the banking deposits of the South Asian countries. This study aims to find the systemic risk spillover between the stock markets and the banking deposits of South Asian countries. We employ the DCC GARCH model to achieve the objective. There are four Asian stock markets and banking deposits used in the study (India, Nepal, Pakistan, and Sri Lanka). The study establishes the presence of a systemic risk spillover between the stock markets and the banking deposits of South Asian countries. The results show a significant positive impact of coefficients between the stock market of India and their banking deposits. The results of Nepal and Pakistan show that theta (1) has a positive significant impact and theta (2) has a significant negative impact between stock markets and banking deposits. The results for Sri Lanka show that theta (1) positively impacts the relationship between the stock market and banking deposits. The theta (2) shows the insignificant negative impact between the stock market and banking deposits. The relationship between theta 1 and theta 2 is negatively correlated with Pakistan, Nepal, and Sri Lanka which means there is less systemic risk in the stock market and the banking deposits of these countries whereas the coefficients of India are positively correlated and there is more systemic risk between the stock market and banking deposits of India. If we look at the result of DCC GARCH, which has a stability equation of stability condition: theta (1) + theta (2) < 1. The stability condition in all countries is present in the DCC GARCH model.

This paper finds the association between the bank deposits and stock markets of South Asian countries. This association increases with the spillover of systemic risk. The effect is stronger where the financial sector is highly dependent upon these two sectors. The transmission of risk not only affects these two sectors but also affects the real economy. These two sectors are interrelated in terms of spillover of volatility, thus affecting the sustainability of the whole financial sector. Due to the transmission of fluctuations in the stock market through the deposit channel, the firms that are equity and lending-dependent are more likely to be financially constrained which is detrimental to the sustainable financial sector.

The findings indicate systemic risk spillover between the stock markets and banking deposits of South Asian countries. This information benefits investors who make investment decisions in these countries because the diversification benefits in these two investments are not enough due to the systemic spillover. According to the findings of this research, the systemic risk spillover between the stock markets and banking deposits in India, Pakistan, and Nepal has reached an absolute scale. It is essential for developing stock markets (in South Asia) to streamline their economic and financial systems to reduce their exposure to financial risk. Investors may use the findings to monitor the stock market’s health and make more informed decisions about their sustainable investments in deposits and stock markets of South Asian countries. Policymakers should devise a standard to mitigate the impact of transmission of fluctuations in the stock market through the banking channel to have a sustainable financial sector. The findings may have implications for the role played by the banks and stock markets in resource allocation in a more sustainable way.

### 5.1 Recommendations

The findings indicate systemic risk spillover between the stock markets and banking deposits of South Asian countries. This information benefits investors who make investment decisions in these countries because the diversification benefits in these two investments are not enough due to the systemic spillover. According to the findings of this research, the systemic risk spillover between the stock markets and banking deposits in India, Pakistan, and Nepal has reached an absolute scale. It is essential for developing stock markets (in South Asia) to streamline their economic and financial systems to reduce their exposure to financial risk. Investors may use the findings to monitor the stock market’s health and make more informed decisions about their sustainable investments in deposits and stock markets of South Asian countries. Policymakers should devise a standard to mitigate the impact of transmission of fluctuations in the stock market through the banking channel to have a sustainable financial sector. The findings may have implications for the discussion of the role played by the banks and stock markets in resource allocation in a more sustainable way.

## Supporting information

S1 Dataset (XLSX)
